# Treatment Patterns and Healthcare Utilization on Pediatric Atopic Dermatitis With Allergic Comorbidities: A Japanese Claims‐Based Study

**DOI:** 10.1111/1346-8138.70102

**Published:** 2026-01-08

**Authors:** Masaki Futamura, Yumi Kang, Ambrish Singh, Junichi Danjo, Takashi Matsuo, Hitoe Torisu‐Itakura, Mizuho Nagao

**Affiliations:** ^1^ Department of Pediatrics NHO Nagoya Medical Center Aichi Japan; ^2^ Eli Lilly Japan K.K. Hyogo Japan; ^3^ Eli Lilly and Company Lilly Bengaluru Bengaluru Karnataka India; ^4^ Department of Clinical Research NHO Mie National Hospital Tsu Japan

**Keywords:** atopic dermatitis, comorbidities, healthcare costs, healthcare resource utilization, treatment patterns

## Abstract

Atopic dermatitis (AD) is a chronic, itchy skin disease that often begins in infancy and may persist into adulthood. The high co‐occurrence of allergic comorbidities (ACMs; asthma, food allergy, and allergic rhinitis) makes it a growing public health concern. However, real‐world data on treatment patterns and the economic burden of AD in children remain sparse. This retrospective observational study aimed to assess the clinical profiles of pediatric patients with AD using data from the JMDC Claims database. Children aged 0–6 years diagnosed with AD between January 2018 and September 2023 were included. A total of 244 316 children with AD (mean age: 3.1 years; 51.3% male) were included. Of these, 17.7% had AD‐only, and 82.3% had AD with ACM. Allergic rhinitis was the most prevalent ACM. Topical corticosteroids were the most prescribed treatment, with 94.0% of patients with ACMs and 85.5% of those with AD‐only receiving them. Potent corticosteroids were more frequently used in the AD with ACM group. Systemic steroids (31.5% vs. 4.8%) and antihistamines (95.5% vs. 56.1%) were used more often in the AD with ACMs group than in the AD‐only group. Patients in the AD with ACM versus AD‐only group had more outpatient visits (11.1/year vs. 6.5/year) and comparable hospitalization frequency, but shorter hospital stays (2.4 vs. 7.7 days per year). Median annual healthcare costs were substantially higher in the AD with ACM group compared to the AD‐only group (139 391 Yen vs. 98 646 Yen), with costs increasing as the number of ACMs increased. Notably, 84.7% of patients with three ACMs incurred annual healthcare costs exceeding 100 000 Yen. These findings highlighted the increased clinical and economic burden associated with the increasing number of ACMs in children with AD, emphasizing the need for more intensive treatment and healthcare resources.

## 1 Introduction

Atopic dermatitis (AD) is a chronic, itchy skin condition with eczematous lesions and immune‐mediated inflammation [1, 2]. It begins mostly in infancy but may persist into adulthood characterized by a relapsing–remitting course, wherein symptoms are in cycles of flare‐up and periods of remission [3]. In infancy, AD also initiates the “atopic march”, a progression to other allergic diseases such as asthma, food allergy, and allergic rhinitis [4, 5]. This progression imposes a substantial clinical burden on children with AD, as these diseases often co‐occur with AD as allergic comorbidities (ACMs), further complicating disease management and outcomes [6, 7, 8].

AD significantly impacts both pediatric patients and their caregivers, causing persistent discomfort, and necessitating frequent medical visits and hospitalizations, which increases the healthcare burden and long‐term costs. The development of ACMs further intensifies disease burden and increases healthcare utilization, placing additional financial strain on families and the healthcare system [9, 10]. Although new drugs for AD are emerging [11], they may not adequately address the treatment of ACMs. Despite the high prevalence of AD with ACMs in children [4, 12, 13], evidence on treatment patterns, and the associated economic burden remains limited. Evidence from large‐scale real‐world data in this population is essential.

In this study, we investigated the clinical profiles of pediatric patients (0–6 years) with AD and evaluated the impact of ACM on treatment patterns and the healthcare resource utilization (HCRU) using data from the JMDC database.

## 2 Methods

### 2.1 Data Source

The JMDC database is a Japanese nationwide claims database from JMDC Co. Ltd. (Tokyo, Japan), which uses standardized disease classifications and anonymous record linkage, and contains data from approximately 19 million Japanese individuals as of June 2024. It contains de‐identified information from inpatient, outpatient, dispensing records, and medical examination data pooled from various health insurance associations [14, 15].

### 2.2 Study Design and Study Population

This retrospective observational study analyzed the characteristics of pediatric patients with AD, including the progression of comorbidities, treatment patterns, and HCRU. Figure S1 depicts the study design. Data were extracted for children aged between 0 and 6 years (inclusive; on the index date) with a confirmed diagnosis of AD (International Classification of Diseases‐10th revision WHO2013 Code L20: L20.0, L20.8, and L20.9) during the study period from January 1, 2018 to September 30, 2023. The index date was the earliest date of AD diagnosis during the study period. Patients were followed from the index date until the earliest occurrence of death, the end of the study period, or loss to follow‐up.

According to the Japanese Ethical Guidelines for Medical and Health Research Involving Human Subjects [16], this study did not require ethical review and informed consent because of its non‐interventional, retrospective study design utilizing anonymized patient data.

### 2.3 Outcomes

#### 2.3.1 Patient Characteristics

We documented all demographic characteristics in each patient's claims data set. The comorbidities including ACMs were recorded during the pre‐defined length of the period after and on the index date.

#### 2.3.2 Treatment Patterns

All treatments related to AD prescribed during the follow‐up periods were reported as frequencies and percentages. Treatment patterns were classified into three categories as topical treatments, systemic treatments, and procedures. Topical treatments included topical corticosteroids (TCS), non‐steroidal treatments such as Janus kinase (JAK) inhibitors, calcineurin inhibitors, phosphodiesterase 4 (PDE‐4) inhibitors, and other treatments like moisturizers. Systemic treatments comprised antihistamines, systemic steroids, ciclosporin, oral JAK inhibitors, and biologics. Procedures referred specifically to phototherapy.

Patients were considered to have received TCS treatment if they were prescribed TCS at least once during the study period. Patients could be included in multiple categories based on the ranks of TCSs they received. All prescribed TCSs were categorized and ranked according to the Japanese clinical guidelines [17].

#### 2.3.3 Healthcare Resource Utilization

Total healthcare costs and HCRU were calculated annually per person. All‐cause HCRU included the following components: Frequency of emergency room and outpatient visits, and hospitalizations. Hospitalization patterns, including the frequency and duration of hospital stays, were also analyzed.

### 2.4 Statistical Analysis

This study was designed to generate descriptive data to understand the characteristics of pediatric patients with AD and their treatment patterns in recent years, and no statistical comparisons were made between the groups of interest. No formal statistical hypothesis was tested; hence, no formal sample size calculations were performed.

Patient profiles were categorized into two subgroups: The AD‐only group, comprising patients without any ACM (food allergy, allergic rhinitis, or asthma) and the AD with ACM group, including patients who had one or more of these ACMs. Patients were monitored for the development of ACMs throughout the follow‐up period. Outcomes were analyzed based on these two subgroups.

Descriptive statistics were used to summarize all variables: Means and standard deviations (SD), or median (inter quartile range [IQR]) for continuous variables, and frequencies and percentages for categorical variables. HCRU data were further adjusted annually.

Systemic steroids use was analyzed in patient groups with AD, comparing those with and without asthma. TCS use was evaluated in relation to the number of ACMs and classified according to potency/strength categories. Additionally, TCS use was analyzed by age group, comparing patients under 3 years of age with those aged 3–6 years.

Healthcare cost data were analyzed for patients with AD accompanied by 1, 2, or 3 ACMs. Annual healthcare expenditures were further analyzed by categorizing annual expenses into those above and below 100 000 Yen, which is the average annual medical cost per child in Japan [18]. To derive the annual cost, each patient's follow‐up period was converted to years to account for variations in follow‐up duration.

Statistical analysis was performed using the Instant Health Data (IHD) platform (Panalgo, Boston, MA). Statistical tests for between‐group comparisons were not conducted.

## 3 Results

### 3.1 Patient Characteristics

**FIGURE 1:**
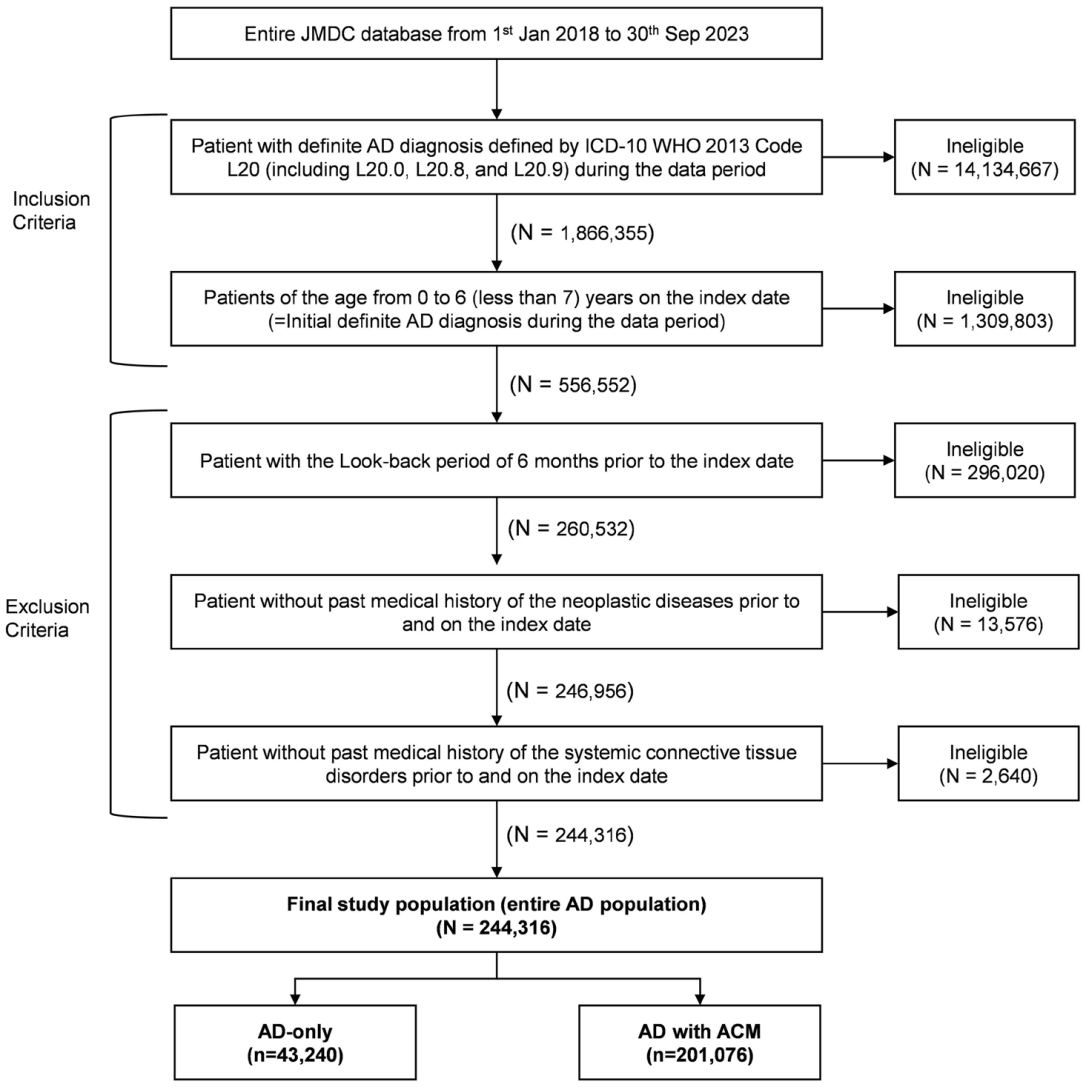
Patient selection flow. ACM, associated comorbidities; AD, atopic dermatitis; *n*, number of patients; *N*, number of records available.

**FIGURE 2:**
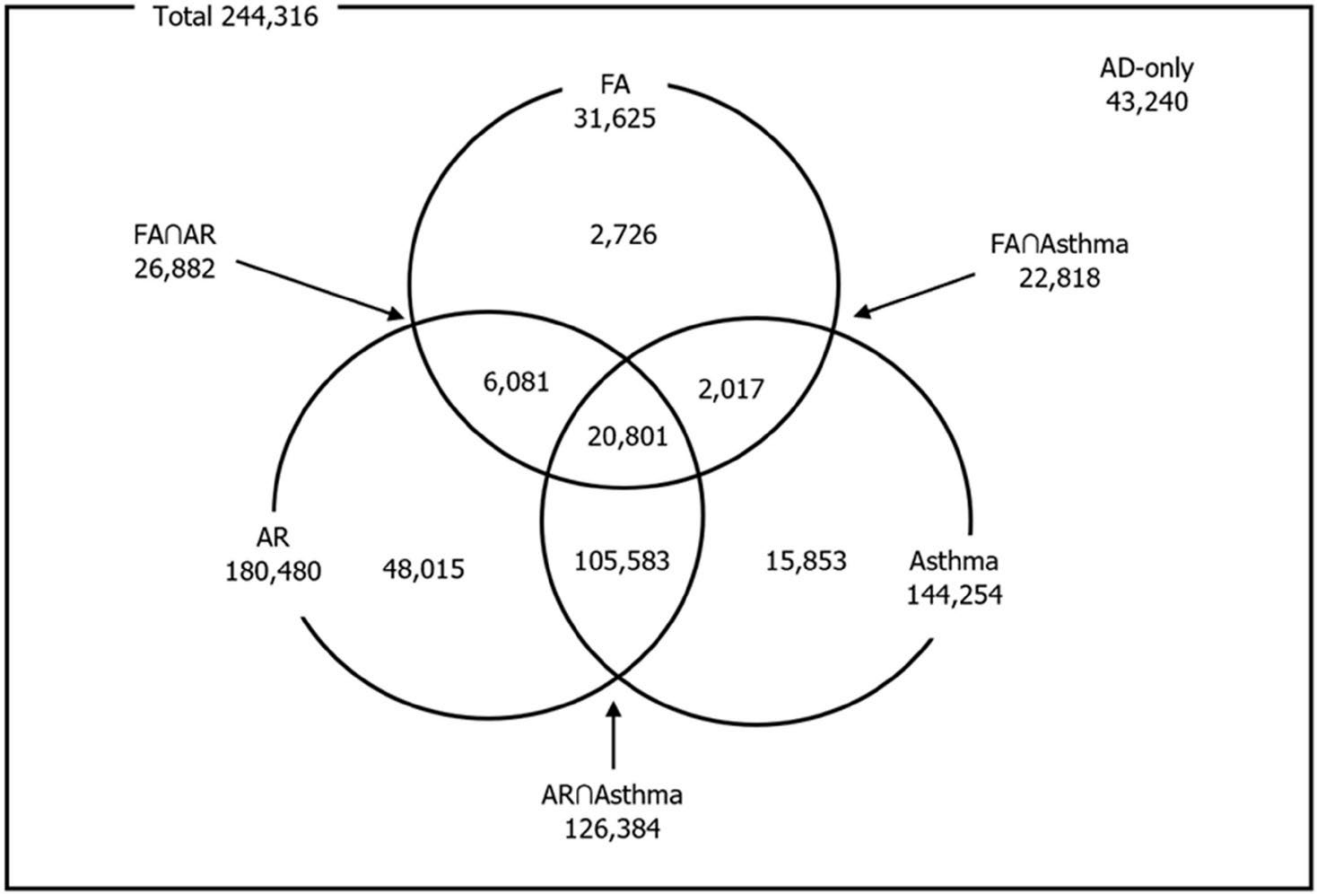
Patient distribution in the AD with ACM. The total number of AD patients (*N* = 244 316) was used as the denominator for calculating percentages. ACM, associated comorbidities; AD, atopic dermatitis; AR, allergic rhinitis; FA, food allergy.

The JMDC claims database recorded 556 552 patients aged 0–6 years diagnosed with AD, between 2018 and 2023. Of these, 244 316 patients with continuous enrolment ≥ 6 months before the index date was included in the study. Among those included, 43 240 (17.7%) patients had AD‐only, while 201 076 (82.3%) had AD with any ACM (Figure 1). Including those diagnosed during the follow‐up period, 180 480 (73.9%) patients with AD had allergic rhinitis, 144 254 (59.0%) had asthma, and 31 625 (12.9%) had food allergies (Figure 2).

In the overall cohort, the mean age was 3.1 (SD 2.0) years, with 51.3% of patients being male. However, females were more prevalent in the AD‐only group (52.5%). The mean follow‐up duration after the diagnosis of AD was 2.6 (SD 1.6) years. Patients in the AD with ACM group were followed for a longer period than those in the AD‐only group, 2.8 and 1.5 years, respectively. A greater proportion of patients in the AD with ACM group (84.3%) were followed for at least 1 year, compared with 46.7% in the AD‐only group. The medical history of patients, excluding ACMs, included chronic eczema, urticaria, and conjunctivitis (Table S1).

### 3.2 Treatment Patterns

#### 3.2.1 Topical Treatment

**FIGURE 3:**
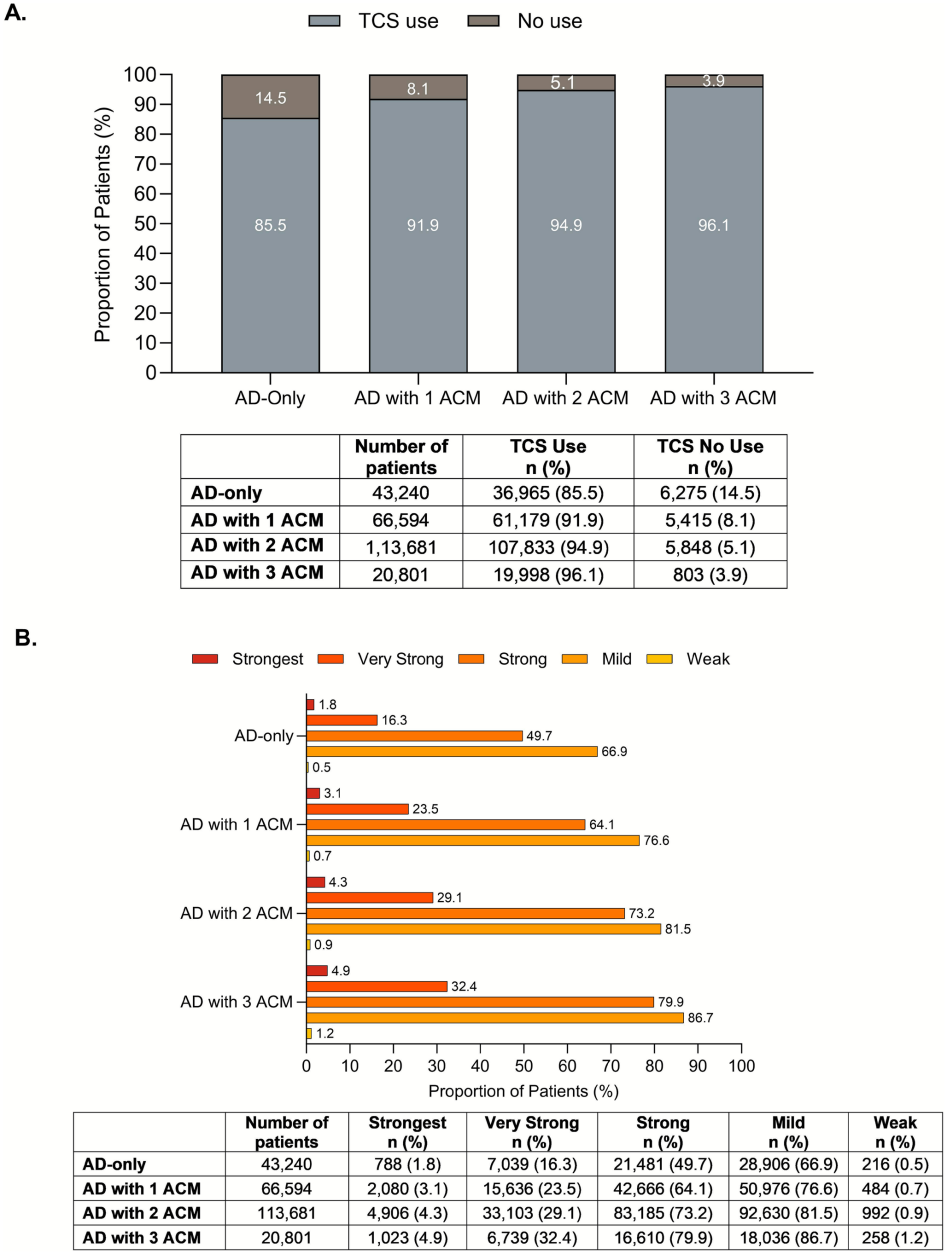
TCS use in patients with AD‐only and in patients with AD with ACM. (A) Patients with any topical steroid use versus no use. (B) TCS use by potency ranks across subgroups—AD‐only, and AD with 1, 2, or 3 ACMs (number of prescriptions per year). ACM, associated comorbidities; AD, atopic dermatitis; *n*, number of patients; TCS, topical corticosteroid. Rank 1: Strongest; Rank 2: Very strong; Rank 3: Strong; Rank 4: Medium/mild; Rank 5, weak.

Topical treatments were the most commonly used, with over 90% of patients with AD (total AD group) using topical corticosteroids, followed by moisturizers or emollients. Compared with the AD‐only group, more patients in the AD with ACM group used TCS (Table 1). There was a clear upward trend in TCS usage as the number of ACM increases (Figure 3A). Across all potency ranks, TCS were more frequently prescribed in patients of the AD with ACM group compared to those of the AD‐only group: strongest rank (4.0% vs. 1.8%), very strong rank (27.6% vs. 16.3%), strong rank (70.9% vs. 49.7%), and mild rank (80.4% vs. 66.9%). TCS were most frequently used in patients with more ACMs, regardless of their potency ranking (Figure 3B).

**TABLE 1:** Treatment patterns in patients with AD‐only and in patients with AD with ACM.

Parameter	Total AD	AD‐only	AD with ACM
Number of patients	244 316	43 240	201 076
Topical treatments, *n* (%)			
Topical corticosteroids	225 975 (92.5)	36 965 (85.5)	189 010 (94.0)
Non‐steroidal topical treatments			
JAK inhibitors	19 075 (7.8)	3771 (8.7)	15 304 (7.6)
Calcineurin inhibitor	18 697 (7.7)	2193 (5.1)	16 504 (8.2)
PDE‐4 inhibitor	7026 (2.9)	1360 (3.2)	5666 (2.8)
Other topical treatments			
Moisturizers or emollients	213 191 (87.3)	33 770 (78.1)	179 421 (89.2)
Systemic treatments, *n* (%)			
Antihistamines	216 227 (88.5)	24 234 (56.1)	191 993 (95.5)
Systemic steroids	65 448 (26.8)	2086 (4.8)	63 362 (31.5)
Ciclosporin	109 (0.0)	11 (0.0)	98 (0.1)
JAK inhibitors	1 (0.0)	0 (0.0)	1 (0.0)
Biologics	0 (0.0)	0 (0.0)	0 (0.0)
Procedure, *n* (%)			
Phototherapy	5390 (2.2)	523 (1.2)	4867 (2.4)

Abbreviations: ACM, associated comorbidities; AD, atopic dermatitis; JAK‐I, Janus kinase inhibitor; *n*, number of patients; NSAID, non‐steroidal anti‐inflammatory drug; PDE‐4, phosphodiesterase‐4.

TCS use was also comparable between children under 3 years and those aged between 3 and 6 years. Potent corticosteroids (strongest rank, very strong rank, and strong rank) were more frequently prescribed in the 3 to 6‐year age group than in those under 3 years. This age‐related prescribing pattern was consistently observed across both patient subgroups: those with AD‐only and those with AD and ACM (Table S2).

#### 3.2.2 Systemic Treatments

Antihistamines were the most frequently used among systemic treatments, followed by systemic steroids. Examining the subgroups, 95.5% of patients in the AD with ACM group were prescribed antihistamines, compared with 56.1% in the AD‐only (Table 1).

Systemic steroids were prescribed to 65 448 (26.8%) patients with AD during the follow‐up period. Among them, 63 362 (31.5%) patients in the AD with ACM group received systemic steroids, compared to 2086 (4.8%) patients in the AD‐only group (Table 2). The number of annual prescriptions of systemic steroids was also higher in the AD with ACM group (0.7 ± 2.5) compared to the AD‐only group (0.2 ± 3.3). Overall, more than 3 systemic steroids were used by 11 491 (4.7%) patients, including 444 patients (1.0%) in the AD‐only group and 11 047 patients (5.5%) in the AD with ACM group. The use of systemic steroids was notably higher in patients having AD with asthma compared to those without asthma (Table 2).

**TABLE 2:** Systemic steroid use in patients with AD‐only and in patients with AD and ACM (number of prescriptions per year).

Parameter	Total AD (*n* = 244 316)	AD‐only (*n* = 43 240)	AD with ACM (*n* = 201 076)	AD with asthma (*n* = 144 254)	AD without asthma (*n* = 100 062)
Patient with any systemic steroid use, *n* (%)	65 448 (26.8)	2086 (4.8)	63 362 (31.5)	52 640 (36.5)	12 808 (12.8)
**Number of annual systemic steroid use, *n* (%)**					
1	34 985 (14.3)	1085 (2.5)	33 900 (16.9)	28 067 (19.5)	6918 (6.9)
2	13 147 (5.4)	378 (0.9)	12 769 (6.4)	10 658 (7.4)	2489 (2.5)
3	5825 (2.4)	179 (0.4)	5646 (2.8)	4687 (3.3)	1138 (1.1)
> 3	11 491 (4.7)	444 (1.0)	11 047 (5.5)	9228 (6.4)	2263 (2.3)

Abbreviations: ACM, associated comorbidities, AD, atopic dermatitis; *n*, number of patients.

A small percentage of patients received phototherapy, with 1.2% in the AD‐only group and 2.4% in the AD with ACM group (Table 1).

### 3.3 Healthcare Resource Utilization

Overall, 23 110 patients (11.5%) in the AD with ACM group experienced one or more hospitalizations, compared to 1447 (3.4%) in the AD‐only group. The hospitalization frequency was comparable in both groups. The average length of hospital stay was longer in the AD‐only group than in the AD with ACM group (7.7 vs. 2.4 days). The frequency of annual emergency room visits was higher in the AD‐only group, with a mean of 1.08 visits per patient, compared to 0.83 visits in the AD with ACM group. In contrast, annual outpatient visits were more frequent in the AD with ACM group (11.1 visits) than in the AD‐only group (6.5 visits) (Table 3).

**TABLE 3:** Healthcare resource utilization pattern.

Parameter	Total AD (*n* = 244 316)	AD‐only (*n* = 43 240)	AD with ACM (*n* = 201 076)
Hospitalization			
Patients with record of hospitalizations, *n* (%)	24 557 (10.1)	1447 (3.4)	23 110 (11.5)
Frequency of annual hospitalizations, mean ± SD	0.1 ± 0.53	0.1 ± 0.75	0.1 ± 0.46
Length of hospitalization, mean days ± SD	2.7 ± 7.7	7.7 ± 22.8	2.4 ± 5.3
Length of hospitalization (patient), *n* (%)			
< 1 week	22 932 (9.4)	1151 (2.7)	21 781 (10.8)
1–2 weeks	254 (0.1)	41 (0.1)	213 (0.1)
> 2 weeks	1319 (0.5)	254 (0.6)	1065 (0.5)
Emergency room visit			
Patients with record of ER visits, *n* (%)	45 318 (18.6)	3794 (8.8)	41 524 (20.7)
Frequency of annual ER visits, mean ± SD	0.9 ± 1.8	1.1 ± 1.7	0.8 ± 1.8
Outpatient visit			
Patients with record of outpatient visits, *n* (%)	237 467 (97.2)	36 422 (84.3)	201 045 (100.0)
Frequency of annual outpatient visits, mean ± SD	10.3 ± 6.1	6.5 ± 5.6	11.1 ± 5.9

Abbreviations: ACM, associated comorbidities; AD, atopic dermatitis; ER, emergency room; *n*, number of patients; SD, standard deviation.

**FIGURE 4:**
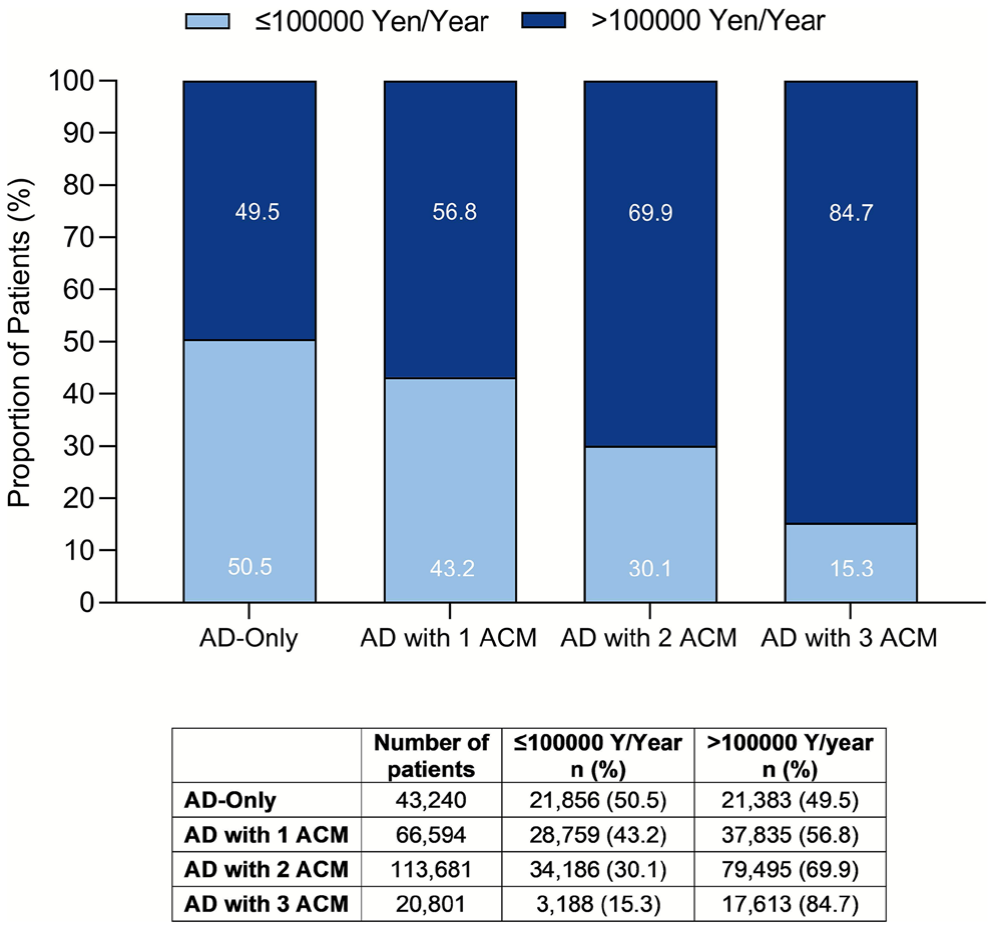
Total healthcare cost (Yen) per patient per year. ACM, associated comorbidities; AD, atopic dermatitis; *n*, number of patients; Y, Yen.

The median annual healthcare cost per pediatric patient with AD was 132 691 Yen/patient. The median annual cost was higher in the AD with ACM group (139 391 Yen/patients) compared to the AD‐only group (98 646 Yen/patient). The analysis examining the cost impact of ACMs found that the AD with more ACMs was associated with higher annual healthcare costs (AD with 3 ACM: 193671 Yen/patient vs. AD with 2 ACM: 144813 Yen/patient vs. AD with 1 ACM: 114741 Yen/patient) (Table 4). This trend remained consistent when assessing the proportion of patients with high expenditures, with a greater percentage of patients in the group of patients with more ACMs incurring annual healthcare costs exceeding 100 000 Yen/patient (Figure 4). Notably, patients with AD and asthma had a higher annual median healthcare cost (150 618 Yen/patient) than those without asthma (107 190 Yen/patient).

**TABLE 4:** Total healthcare cost (Yen) for patient per year.

Parameter	Total AD (*n* = 244 316)	AD‐only (*n* = 43 240)	AD with ACM (*n* = 201 076)	AD with ACM (*n* = 201 076)		
				AD with 1 ACM (*n* = 66 594)	AD with 2 ACMs (*n* = 113 681)	AD with 3 ACMs (*n* = 20 801)
Total healthcare cost (Yen/person/year), median	132 691	98 646	139 391	114 741	144 813	193 671
IQR	77 263–221 815	50 497–188 699	83 798–227 130	67 281–194 411	89 808–231 106	125 759–294 846

Abbreviations: ACM, associated comorbidities; AD, atopic dermatitis; IQR, interquartile range.

## 4 Discussion

This study provided valuable insights into the clinical, treatment, and economic burden associated with pediatric patients with AD in Japan, utilizing the JMDC database. Our findings highlight notable differences in treatment approaches, healthcare utilization, and associated costs between patients with AD only and those with AD and ACMs.

One of the key findings of this study was that a substantial proportion of pediatric patients with AD also exhibited one or more ACMs. Allergic rhinitis was the most frequently observed ACM, followed by asthma and food allergies. These results were consistent with patterns of comorbidities in children with AD reported in previous studies, reinforcing the concept of the “atopic march” [19, 20]. This concept suggests that AD represents the initial clinical manifestation of the allergic disease spectrum including allergic rhinitis, asthma, and food allergy. These are well‐recognized comorbidities of AD and their prevalence increases in parallel with the severity of AD [4, 8, 19, 21, 22, 23, 24].

The frequent occurrence of ACMs among pediatric patients with AD complicates treatment regimens [6, 7, 8, 9]. The treatment patterns observed in this study highlighted a substantial reliance on TCS, irrespective of ACM status. TCS were the most commonly prescribed treatment and reaffirmed the pivotal role in managing both patients with AD‐only and AD with ACMs. These findings supported the previous real‐world evidence, which reported that the use of potent TCS increased particularly in patients with more severe AD from 2005 to 2017 in Japan [25]. This coincides with the period during which Japanese clinical guidelines have recommended TCS as the primary treatment option [17, 26].

Notably, the use of TCS across all potency ranks was consistently higher in the AD with ACMs group compared to the AD‐only group. Moreover, TCS use increased progressively with the number of ACMs. This trend suggests that these comorbidities exacerbated AD symptoms and necessitated the use of stronger corticosteroids [3, 7]. This reinforces the need for tailored treatment plans for pediatric patients with AD to manage both their underlying condition and the comorbidities effectively. While age‐specific trends in TCS use revealed similar patterns across the two age groups, stronger TCS were more frequently used in patients aged 3–6 years. This may reflect the higher proportion of moderate‐to‐severe cases that were in the older age group. It also suggests that weaker corticosteroids may be preferred for infant patients more often than necessary, while the clinical guidelines recommend using the appropriate TCS according to the severity of eczema regardless of the patient's age [17, 26, 27].

In the current study, a higher proportion of patients in the AD with asthma subgroup received systemic steroids. This may be due to their common use in treating acute asthma exacerbations. Notably, a significant number of patients in the AD without asthma group also received systemic steroids, though it is not recommended in the clinical guideline. Pediatricians generally refrain from the prolonged use of systemic steroids in pediatric patients because of potential adverse effects on growth [28, 29]. Nevertheless, the decision to use systemic steroid therapy might reflect the substantial burden that AD imposes on both patients and their caregivers.

Longer mean follow‐up duration observed in the AD with ACMs group reflected the chronic nature of ACM. The frequency of outpatient visits was higher in patients who had AD with ACMs. This indicated a higher demand for regular medical care and a greater burden in managing the disease with increasing severity [9]. Although patients in the AD‐only group experienced fewer hospitalizations, they had more emergency room visits, and longer hospital stays. The small number of hospitalized patients (*n* = 1447) and the high variability in length of stay (mean: 7.7 days; SD: 22.8) suggest diverse healthcare needs. This may likely reflect admissions for acute episodes related to non‐AD conditions or comorbidities requiring more intensive monitoring and diagnostics [17]. Additionally, some patients may have undergone “educational hospitalization,” a 2‐week stay aimed at learning to manage AD effectively [30, 31]. These findings should be interpreted with caution due to the small sample size in the AD‐only group, which may have influenced the observed patterns.

Annual healthcare costs were substantially higher for patients who had AD with ACMs compared to those with AD‐only. It highlights the increased disease complexity and healthcare utilization in the presence of ACMs. To our knowledge, there were no previous nationwide studies investigating the annual healthcare costs for pediatric AD. Our findings align with a previous web‐based self‐reported survey in Japanese adults, which revealed increasing annual expected medical costs in adults with AD as disease severity progressed [32].

Our analyses suggest an association between the number of ACMs and increased healthcare burden. In the group with a higher number of ACMs, the median annual healthcare cost was higher, and the proportion of patients with medical costs above average was also higher. This trend aligns with previous studies suggesting that patients with multiple comorbidities typically incur greater healthcare expenditures [10, 18, 33]. Overall, our findings underscore the incremental financial burden of managing AD with an increasing number of ACMs. Given the higher frequency of outpatient visits and hospitalizations in the AD with ACM group, healthcare costs may also serve as an indicator of disease burden. These insights highlight the importance of intervention for ACMs in pediatric AD to mitigate long‐term healthcare costs.

This study has some limitations that should be considered when interpreting the findings. First, patients with AD and ACMs were identified based on at least one recorded diagnosis code in the claims database. While this approach aligns with standard practices in real‐world evidence research, claims‐based diagnoses may not always reflect the true clinical condition. Second, prescription records derived from claims data do not guarantee actual medication usage. Physicians prescribed medications based on how severe the disease was, and we believe that the strength of the TCS used was directly related to the severity of the AD. Third, the JMDC database does not include detailed clinical information such as disease severity, other clinical information and the prescription purpose of each medication. As a result, it is unclear whether corticosteroids were used to treat AD or ACMs, but they may have been effective for multiple allergic diseases. Fourth, the JMDC database predominantly includes employees of large Japanese companies, which may not fully represent the broader pediatric population. However, it remains the largest database for hospitalization and outpatient treatment in Japan, enabling longitudinal follow‐up of each patient across different hospitals. Finally, this study was exploratory and did not aim to test specific hypotheses; therefore, only descriptive analyses were conducted, and no statistical comparisons were made between the groups of interest.

## 5 Conclusion

The findings from this study suggest that in pediatric patients (0–6 years old) with AD, the coexistence of ACMs may impact both treatment patterns and HCRU. Patients with AD and ACMs used more potent corticosteroids, had more frequent hospitalizations and emergency department visits, and incurred higher annual medical costs.

Overall, this study highlighted that the compounded burden of managing AD is influenced by the comorbidity of asthma, food allergy and allergic rhinitis. These findings offer valuable insights for considering future treatment and informing healthcare policies for AD.

## Funding

This study was sponsored by Eli Lilly Japan K.K., Hyogo, Japan.

## Ethics Statement

According to the Japanese Ethical Guidelines for Medical and Health Research Involving Human Subjects, this study did not require ethical review and informed consent because of its non‐interventional, retrospective study design utilizing anonymized patient data.

## Conflicts of Interest

M.F. has served as a speaker for AbbVie GK., Pfizer Japan Inc., Sanofi K.K., Maruho Co. Ltd., Torii Pharmaceutical Co. Ltd., and Otsuka Pharmaceutical Co. Ltd. Y.K., A.S., J.D., T.M., H.T.‐I. are full‐time employees and stakeholders of Eli Lilly and Company. M.N. has received lecture fees from Eli Lilly, Sanofi K.K., Torii Pharmaceutical Co., Otsuka Pharmaceutical Co. Ltd., and Maruho Co. Ltd.

## Supporting information


**Table S1:** Patient characteristics.
**Table S2:** TCS use in each subgroup.
**Figure S1:** Study design.

## Data Availability

The data that support the findings of this study are available from JMDC. Restrictions apply to the availability of these data, which were used under license for this study. Data are available from https://www.jmdc.co.jp/en/ with the permission of JMDC.
