# Supplementary material for: Treatment Patterns and Healthcare Utilization on Pediatric Atopic Dermatitis With Allergic Comorbidities: A Japanese Claims‐Based Study
Source: J Dermatol. 2026 Jan 8;53(3):437–46. doi: 10.1111/1346-8138.70102 (PMC12967769; doi:10.1111/1346-8138.70102)
Supplement: Supplementary file 1 — Table S1: Patient characteristics. Table S2: TCS use in each subgroup. Figure S1: Study design. [file JDE-53-437-s001.docx]

**Supplementary Material**

**Treatment patterns and healthcare utilization on pediatric atopic dermatitis with allergic comorbidities: A Japanese claims-based study**

Masaki Futamura,^1^ Yumi Kang,^2*^ Ambrish Singh,^3^ Junichi Danjo,^2^ Takashi Matsuo,^2^ Hitoe Torisu-Itakura,^2^ Mizuho Nagao^4^

^1^Department of Pediatrics, NHO Nagoya Medical Center, Aichi, Japan

^2^Eli Lilly Japan K.K., Hyogo, Japan

^3^Eli Lilly and Company, Lilly Bengaluru, Bengaluru, Karnataka, India

^4^Department of Clinical Research, NHO Mie National Hospital, Tsu, Japan

***Corresponding author:**

Yumi Kang

Eli Lilly Japan K.K., Kobe, Hyogo, Japan

E-mail: [yumi.kang@lilly.com](mailto:yumi.kang@lilly.com)

Phone: +81-80-5682-9286

**Table S1.** Patient characteristics

| **Parameter** | **Total AD** | **AD-only** | **AD with ACM** |
| --- | --- | --- | --- |
| **Patients, n** | 244,316 | 43,240 | 201,076 |
| **Age, mean ± SD** | 3.1 ± 2.0 | 3.1 **±** 2.0 | 3.1 ± 2.0 |
| **Gender, n (%)** |  |  |  |
| Female | 119,085 (48.7) | 22,693 (52.5) | 96,392 (47.9) |
| Male | 125,231 (51.3) | 20,547 (47.5) | 104,684 (52.1) |
| **Follow-up duration (years), mean ± SD** | 2.6 **±** 1.6 | 1.5 **±** 1.4 | 2.8 **±** 1.5 |
| **Follow-up years in categories, n (%)** |  |  |  |
| <1 | 54,535 (22.4) | 23,009 (53.3) | 31,526 (15.7) |
| ≥1 | 189,291 (77.6) | 20,135 (46.7) | 169,156 (84.3) |
| **Medical history, n** |  |  |  |
| Chronic Eczema | 185,633 | 31,934 | 153,699 |
| Urticaria | 58,186 | 9,023 | 49,163 |
| Conjunctivitis | 32,479 | 4,039 | 28,440 |
| Blepharitis | 12,236 | 1,989 | 10,247 |
| Vomiting syndrome ^†^ | 2,843 | 404 | 2,439 |
| Vitiligo | 559 | 111 | 448 |
| Alopecia Areata | 408 | 78 | 330 |
| Diabetes mellitus | 168 | 25 | 143 |
| Osteoporosis or osteopenia | 78 | 12 | 66 |
| Ulcerative colitis | 9 | 3 | 6 |
| Crohn’s disease | 6 | 2 | 4 |
| Eosinophilic esophagitis | 3 | 0 | 3 |
| Celiac disease | 0 | 0 | 0 |
| Kawasaki disease | 0 | 0 | 0 |

ACM, associated comorbidities; AD, atopic dermatitis; n, number of patients; SD, standard deviation

^†^Including any of ketogenic, acetonemic and cyclic vomiting

**Table S2.** TCS use in each subgroup

|  | **Total AD** | | **AD-only** | | **AD with ACM** | |
| --- | --- | --- | --- | --- | --- | --- |
| Age subgroup | <3 years  (n=125,295) | 3-6 years  (n=119,021) | <3 years  (n=22,673) | 3-6 years  (n=20,567) | <3 years  (n=102,622) | 3-6 years  (n=98,454) |
| **TCS use, n (%)** |  |  |  |  |  |  |
| Any rank | 117,436 (93.7) | 108,539 (91.2) | 19,578 (86.4) | 17,387 (84.5) | 97,858 (95.4) | 91,152 (92.6) |
| Strongest rank | 3,730 (3.0) | 5,067 (4.3) | 305 (1.4) | 483 (2.4) | 3,425 (3.3) | 4,584 (4.7) |
| Very strong rank | 27,306 (21.8) | 35,211 (29.6) | 2,795 (12.3) | 4,244 (20.6) | 24,511 (23.9) | 30,967 (31.5) |
| Strong rank | 82,643 (66.0) | 81,299 (68.3) | 10,432 (46.0) | 11,049 (53.7) | 72,211 (70.4) | 70,250 (71.4) |
| Mild rank | 106,432 (85.0) | 84,116 (70.7) | 16,850 (74.3) | 12,056 (58.6) | 89,582 (87.3) | 72,060 (73.2) |
| Weak rank | 1,244 (1.0) | 706 (0.6) | 146 (0.6) | 70 (0.3) | 1,098 (1.1) | 636 (0.7) |

ACM, associated comorbidities; AD, atopic dermatitis; n, number of patients; TCS, topical corticosteroid.

**Figure S1.** Study design


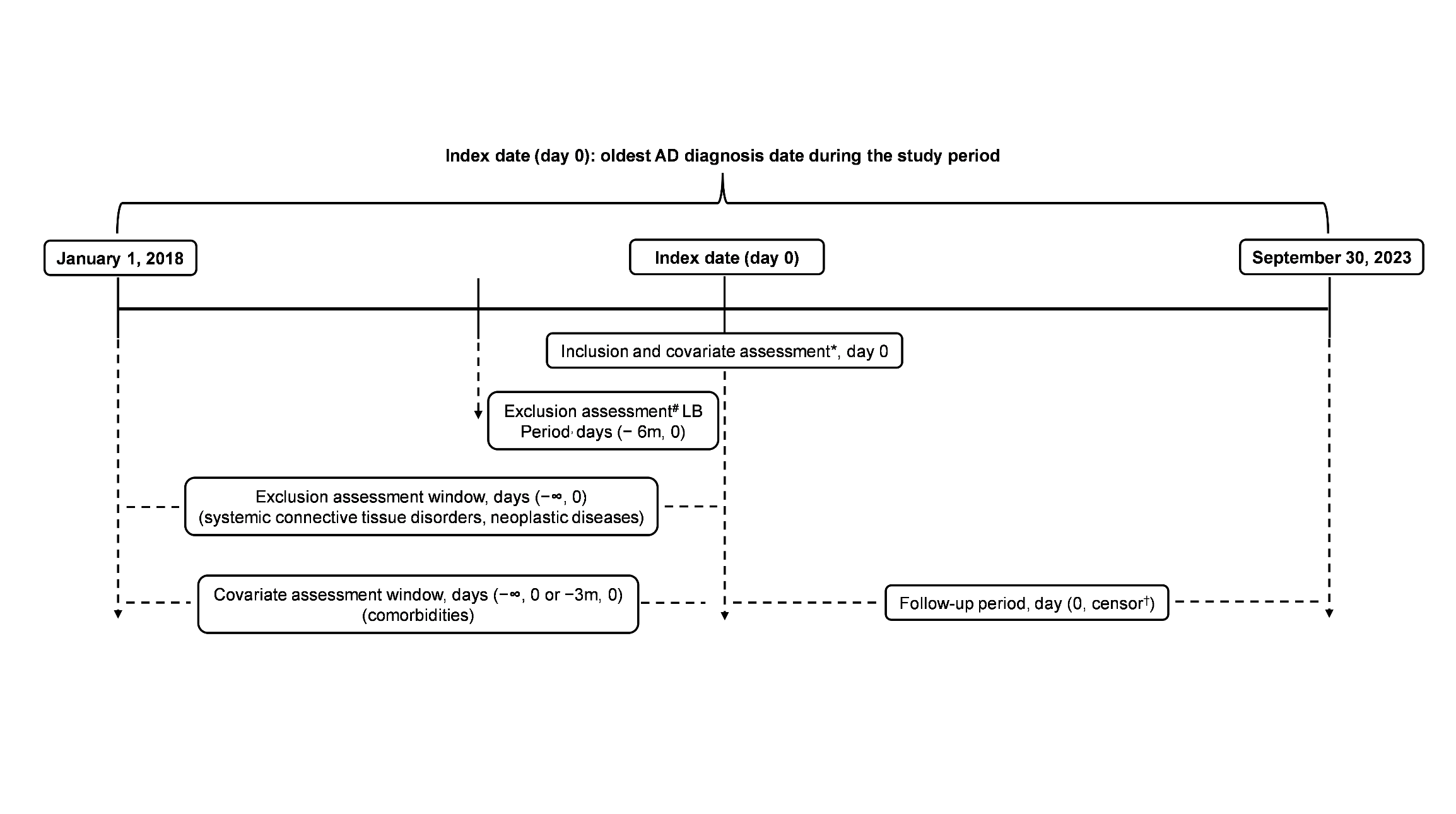


*Inclusion assessment, patient age; covariate assessment, patient characteristics.

^#^LB period of 6 months.

^†^Censoring criteria: earliest occurrence of death, the end of the study period, or loss to follow-up.

ACM, associated comorbidities; AD: atopic dermatitis; LB, look-back; m, month.
